# Religious and Spiritual Influence on Death Stories

**DOI:** 10.1093/geroni/igaf122.1049

**Published:** 2025-12-31

**Authors:** Melanie Evangelista

**Affiliations:** Yeshiva University, New York, New York, United States

## Abstract

While fear and anxiety surrounding death is important to understand, it is only a piece of what shapes one’s perspective on death (Alftberg et al., 2018; Gire, 2014). Death stories, defined as one’s personal narrative encompassing the cumulation of their experiences that shape their view about death, provide individualized and personal insight into one’s often complex thoughts and feelings about death. These stories are influenced by one’s exposure and memory to death and are continually shaped throughout life (Hedtke, 2014). Current literature on spiritual and religious beliefs in relation to thoughts about death indicates that as individuals age, they often become more religious, decreasing fear of death, as fear of death is typically highest among moderately religious individuals and lowest among those who are either nonreligious or extremely religious (Ellis & Wahab, 2013; Jiang et al., 2023; Wink & Scott, 2025). Death and life after death are central tenets of religious and spiritual practices, discussed early in religious education and throughout one’s life. As religion, spirituality and death are intricately intertwined, faith-based language is frequently utilized when discussing and defining one’s thoughts about end-of-life across major world religions (Chakraborty et al., 2017; Gire, 2014). This session seeks to explore how these conversations and experiences contribute to one’s understanding of death, including the influence of religious vocabulary, funerary customs, and after-life beliefs.

